# Dynamic wildlife occupancy models using automated acoustic monitoring data

**DOI:** 10.1002/eap.1854

**Published:** 2019-02-27

**Authors:** Cathleen Balantic, Therese Donovan

**Affiliations:** ^1^ Vermont Cooperative Fish and Wildlife Research Unit Rubenstein School of Environment and Natural Resources University of Vermont 302 Aiken Center, 81 Carrigan Drive Burlington Vermont VT 05405 USA; ^2^ U.S. Geological Survey Vermont Cooperative Fish and Wildlife Research Unit Rubenstein School of Environment and Natural Resources University of Vermont Burlington VT 05405 USA

**Keywords:** automated acoustic monitoring, dynamics, encounter history, false positives, occupancy, parameter bias

## Abstract

Automated acoustic monitoring of wildlife has been used to characterize populations of sound‐producing species across large spatial scales. However, false negatives and false positives produced by automated detection systems can compromise the utility of these data for researchers and land managers, particularly for research programs endeavoring to describe colonization and extinction dynamics that inform land use decision‐making. To investigate the suitability of automated acoustic monitoring for dynamic occurrence models, we simulated underlying occurrence dynamics, calling patterns, and the automated acoustic detection process for a hypothetical species under a range of scenarios. We investigated an automated species detection aggregation method that considered a suite of options for creating encounter histories. From these encounter histories, we generated parameter estimates and computed bias for occurrence, colonization, and extinction rates using a dynamic occupancy modeling framework that accounts for false positives via small amounts of manual confirmation. We were able to achieve relatively unbiased estimates for all three state parameters under all scenarios, even when the automated detection system was simulated to be poor, given particular encounter history aggregation choices. However, some encounter history aggregation choices resulted in unreliable estimates; we provide caveats for avoiding these scenarios. Given specific choices during the detection aggregation process, automated acoustic monitoring data may provide an effective means for tracking species occurrence, colonization, and extinction patterns through time, with the potential to inform adaptive management at multiple spatial scales.

## Introduction

Remote automated acoustic monitoring of sound‐producing wildlife provides a means for characterizing status and trends in species occurrence across landscapes (Cerqueira and Aide 2016). In a typical remote acoustic monitoring program, autonomous recording units (ARUs) are installed at study locations to capture recordings of the environment over time, based on a schedule input to the device by the research team. Vast quantities of audio data may be collected in a short amount of time, from which sounds produced by target monitoring species may be detected. Remote acoustic monitoring offers the potential to efficiently gather occurrence data for sound‐producing wildlife species across regional spatial scales (Furnas and Callas 2015). Long‐term, large‐scale acoustic monitoring programs may be positioned to engage in systematic adaptive management research, wherein continued monitoring reduces uncertainty over time to improve management decisions amid climate change and rapidly changing land uses (Williams et al. 2009).

Characterization of occurrence, or occupancy, requires only species presence–absence data or, more precisely, detection–nondetection data, since the probability of detecting a truly present species (*p*) is often <1, and false negatives transpire when a species is present but not detected (MacKenzie et al. 2002). To characterize false negatives, a site must be surveyed more than once. The fundamental building block of an occupancy model is thus the encounter history, a binary string indicating whether a species was detected or not detected on each survey occasion. Any combination of zeroes and ones is possible; an encounter history of 001, for example, indicates that a site was surveyed three times, and the species was only detected on the third occasion. The original single‐season occupancy model has been expanded upon in several crucial ways, including dynamic (multiple season) models (MacKenzie et al. 2003), models that account for both false negatives and false positives (Royle and Link 2006, Miller et al. 2011, 2013, Chambert et al. 2015), and numerous other advances (Bailey et al. 2014). The dynamic occupancy model is particularly suitable for research seeking to understand trends over time. In addition to initial occupancy status (ψ), this model characterizes local extinction (ε), and colonization (γ) patterns between survey seasons, as well as covariates that influence the initial state and state changes.

Although acoustic monitoring is well suited to capturing dynamic occupancy data for sound‐producing species, the ease and efficiency of acoustic data acquisition can quickly overwhelm research programs with massive audio data streams. To accommodate the ensuing “big data” dilemma, audio recordings may be rapidly processed using computer algorithms for automatically detecting species by their calls. For example, we have used the R package AMMonitor to create customized call templates for target species combined with statistical learning classifiers for this purpose (Katz et al. 2016, Hafner and Katz 2018; C. M. Balantic, T. M. Donovan, *unpublished manuscript*). Numerous other software solutions exist for automated detection, such as Wildlife Acoustics Kaleidoscope (Wildlife Acoustics 2018), Raven Pro (Bioacoustics Research Program 2015), the Arbimon platform (Aide et al. 2013), MatlabHTK (Ranjard et al. 2017), Tadarida (Bas et al. 2017), and Animal Sound Identifier (ASI; Ovaskainen et al. 2018), though none of these provide a streamlined means for aggregating species detection data into occurrence models.

**Figure 1:**
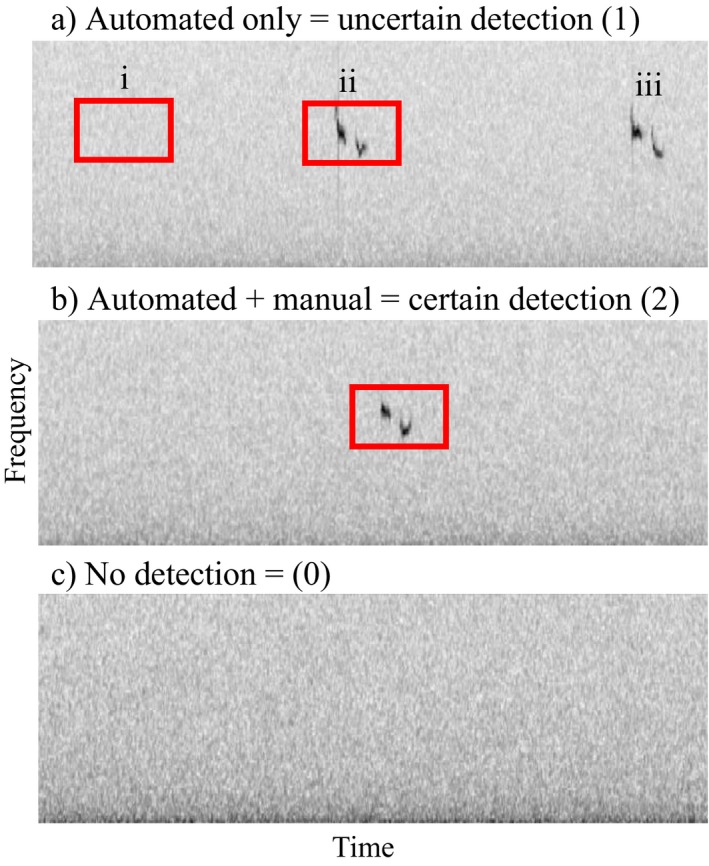
Construction of an encounter history for the Miller model is illustrated with three “surveys” in (a), (b), and (c), represented by audio recording spectrograms. Event‐level detections by a hypothetical automated method for a target species (red boxes) can be event‐level false positives (a‐i) or event‐level true positives (a‐ii). Event‐level false negatives occur where the automated method misses a target signal (a‐iii). Event‐level detections from all recordings within a survey period are collected to produce a single value that describes survey‐level detection status according to the Miller model (0, 1, or 2). (a) Survey ‘a’, which used the automated method only, aggregation produces a ‘1’ in the encounter history to indicate an uncertain detection. (b) Survey ‘b’ underwent a posteriori manual verification; all event‐level detections within this survey are checked by hand. We assign this survey a ‘2’ to denote a certain detection at the survey level. (c) Survey ‘c’ yielded zero event‐level detections and is assigned a ‘0’ at the survey level. Together, surveys a, b, and c produce an encounter history of 120 for the season.

Regardless of the software or detection method used, computer algorithms for automated detection may not detect a species when it is present (“false negative”) or may incorrectly detect an absent species (“false positive”). We take care to distinguish between detection mistakes occurring at the “event level” and those occurring at the “survey level.” Event‐level mistakes are perpetrated by the automated detection method; these occur when the algorithm flags a detection not actually from the target species (“event‐level false positive”; Fig. 1a‐i), or when the algorithm fails to detect an existing signal from the target species (“event‐level false negative” (Fig. 1a‐iii). Event‐level detection mistakes represent a ubiquitous and well‐documented challenge in automated acoustic monitoring research problems (Acevedo et al. 2009, Kalan et al. 2015, Brauer et al. 2016, Katz et al. 2016, Stowell et al. 2016, Shonfield and Bayne 2017). Survey‐level mistakes, on the other hand, originate as a consequence of aggregating event‐level mistakes into an encounter history for occupancy analysis. Ambiguity around how to combine event‐level detections to create survey‐level encounter histories is an area of emergent interest in automated acoustic monitoring and is made especially vexing by acoustic monitoring capacity to generate high numbers of surveys compared with traditional field methods. Robust detection aggregation methodology is paramount for dynamic models, where the consequences of survey‐level detection mistakes are amplified: when false positive detection errors are ignored, estimated extinction and colonization rates can become so biased and imprecise as to render results useless (McClintock et al. 2010, Miller et al. 2015, Ruiz‐Gutierrez et al. 2016).

To address concerns about survey‐level false negatives and false positives, Miller et al. (2013) introduced the “multiple detection states” dynamic occupancy model (hereafter, the Miller model). Survey‐level detection states in the Miller model are categorized as “certain” or “uncertain,” making this framework amenable to cases where humans can later verify a subset of automated detections. In this work, within automated acoustic monitoring contexts, we presume that *all* audio recordings are scanned for the target species using an automated acoustic detection system. Event‐level detections are then aggregated into survey outcomes, which compose the encounter history (Fig. 1). Any survey‐level detections that result via automation only (state 1) are denoted by a 1 to indicate an uncertain detection (Fig. 1a). A subset of surveys, however, is allocated for a posteriori verification, wherein we assume no false positives exist. Surveys with automated event‐level detections corroborated by manual verification (state 2) are given a 2 to represent a certain detection at the survey level (Fig. 1b). A survey containing no event‐level detections is assigned a 0, indicating uncertain absence (Fig. 1c).

To illustrate, the history 120,000 suggests two primary demographic seasons, each surveyed three times. In the first season, assumed closed to demographic change across surveys, the species was detected in the first survey via automation only (producing an uncertain detection), detected with certainty in the second survey via automation with manual verification, and undetected in the third survey. In the second season, also assumed closed to demographic change, the species was not detected in any of the three surveys, all of which connote uncertain absences. The parameters estimated by the Miller model are the initial probability of occupancy (ψ), the state transition probabilities for colonization (γ) and extinction (ε), and a family of detection probability parameters. For uncertain detections, which are acquired via the automated acoustic detection system, *p*_10_ represents the probability of incorrectly detecting the species at an unoccupied site (survey‐level false positive), while *p*_11_ is the probability of correctly detecting a present species at an occupied site (survey‐level true positive). Certain detections are denoted by the parameter *b*, the probability of detecting a species via automated detection paired with manual verification, given presence. The probability of each observed encounter history can be computed for each site given the model parameters, and the product of those probabilities across all sites can be used to estimate parameters with maximum likelihood analysis (Miller et al. 2013).

Although the Miller model may have high utility for acoustic monitoring programs endeavoring to describe local extinction and colonization dynamics, two chief challenges remain. First, minimal guidance exists for aggregating large quantities of event‐level automated acoustic detections into survey‐level encounter histories (though see Chambert et al. 2017, Newson et al. 2017), and we are unaware of any previous efforts to explicitly exploit existing properties of automated detection algorithms for this purpose. Second, it is unclear how encounter history aggregation decisions affect the reliability of the Miller model for producing the unbiased, precise state parameter estimates (ψ, γ, and ε) necessary to make informed monitoring and management decisions. Without a comprehensive framework for moving from audio data collection to mistake‐sensitive dynamic occupancy analysis, acoustic monitoring programs will be hobbled in their capacity to effectively leverage the opportunities offered by long‐term, large‐scale monitoring research, yielding compromised inference about population trends and limited model utility for subsequent adaptive management decisions.

### Objectives

The goal of this paper was to explore methodology for using acoustic data in dynamic occupancy models where observed data may include both false positives and false negatives. Our objectives were to (1) simulate latent occurrence dynamics and calling production, as well as the automated acoustic detection process for a hypothetical target species across *N* sites in a hypothetical monitoring area, (2) introduce an event‐level detection aggregation method that leverages properties of automated acoustic monitoring to create encounter histories under a suite of detection aggregation time frames, detection thresholds, and confirmation capacities, and (3) generate parameter estimates and compute the bias for occurrence, colonization, and extinction rates using the Miller model.

## Materials and Methods

### Simulation of occurrence dynamics, species calling production, and detection process

**Figure 2:**
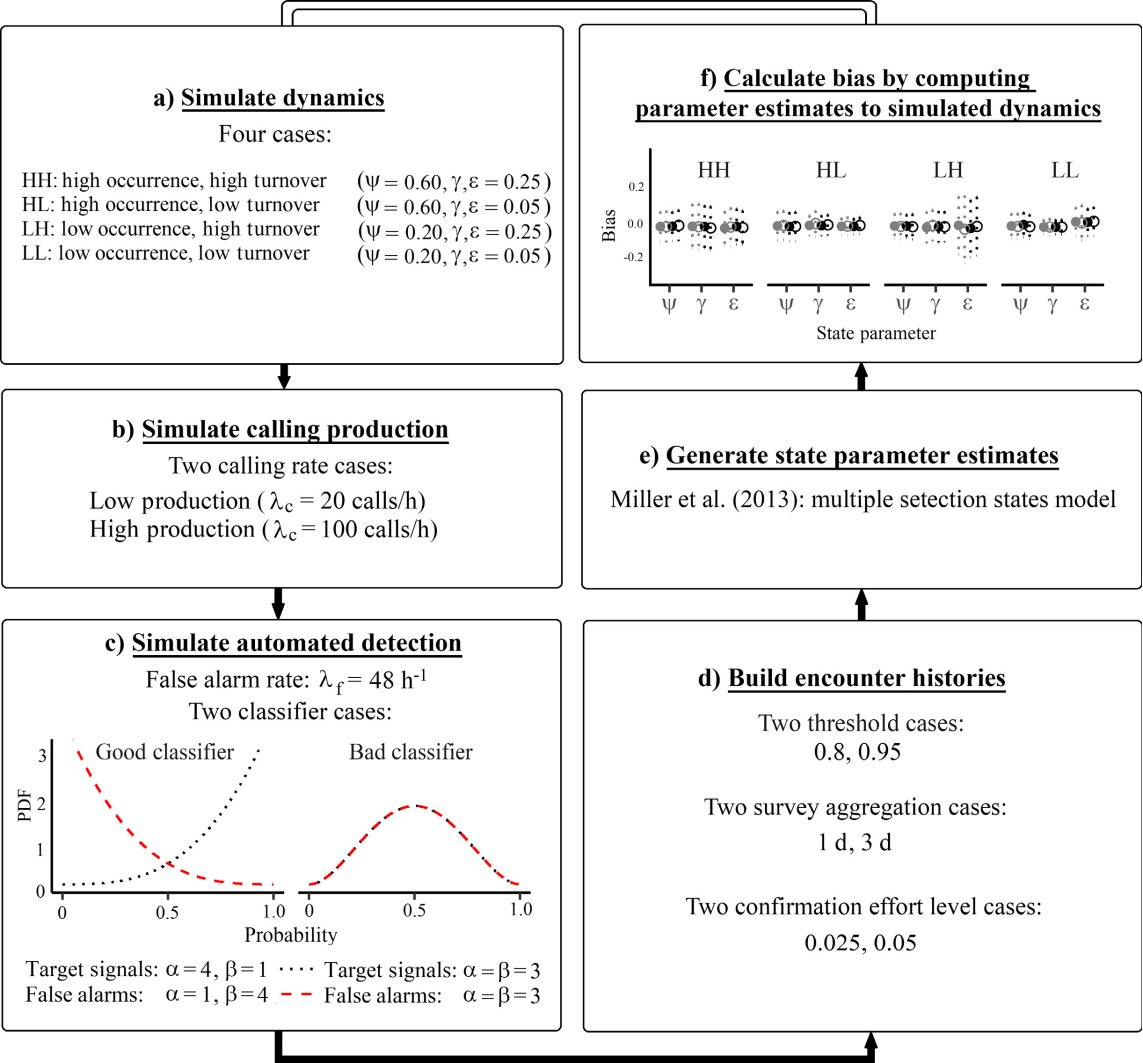
(a) Simulation and parameter estimation workflow for simulating dynamics (parameters are the initial probability of occupancy [ψ], the state transition probabilities for colonization [γ], and extinction [ε]), (b) simulating species calling production (λ_c_ is the average species calling rate per hour), (c) simulating the automated detection process, where the probability distribution function (PDF) is defined by beta distribution shape parameters α and β, (d) building encounter histories, (e) generating parameter estimates, and (f) computing bias.

We simulated two 30‐d seasons of underlying occupancy dynamics and species sound production for a hypothetical target species across 100 sites. We assumed that the 30‐d monitoring period occurred when the species was anticipated to be active and available in the region, whether migratory or not. We also assumed that monitoring sites were selected under a statistical sampling design ecologically meaningful for the hypothetical species with regard to life history characteristics such as territory size and local migratory behavior (i.e., temporal and spatial assumptions of the Miller model were met). For simplicity, we did not use site or survey‐level covariates to model any processes. To create dynamic occupancy scenarios, we used the four simulation cases outlined by Miller et al. 2015 (Fig. 2a): high initial occurrence with high turnover (HH; ψ = 0.60, γ = ε = 0.25), high initial occurrence with low turnover (HL; ψ = 0.60, γ = ε = 0.05), low initial occurrence with high turnover (LH; ψ = 0.20, γ = ε = 0.25), and low initial occurrence with low turnover (LL; ψ = 0.20, γ = ε = 0.05).

For each dynamic occupancy scenario, we simulated the underlying sound production process wherever the species was present (Fig. 2b). Individual calling rates vary widely based on species, breeding stage, and environmental conditions (Catchpole and Slater 2008), with overall abundance driving the total number of target signals available in the soundscape (Royle and Nichols 2003). We condensed these elements into an average species calling rate per hour, λ_c_, and investigated two cases: (1) a low call production scenario that averaged 20 calls/h (or 0.33 calls/min; λ_c_ = 20), and (2) a high call production scenario that averaged 100 calls/h (or 1.67 calls/min; λ_c_ = 100). For each sampled minute of the season, we used a Poisson process to generate the true number of calls produced by the species.

Next, we simulated the automated acoustic detection process (Fig. 2c) where we addressed three components: (1) the timing and frequency of audio recordings, (2) the existence of “false alarm” sources within the recording soundscape, and (3) the general aptitude of the automated detection method. First, we chose a recording scheme of five minutes of audio sampling per day, presumed to occur during ideal windows for capturing target species call production. Second, we used a Poisson process to inject false alarms (λ_f_) into each minute of audio recording. The false alarm rate, λ_f_, acts as an analog to the call production rate (λ_c_), in that it connotes underlying sources of false alarms present in the soundscape, which fool an automated detector into generating event‐level false positives. We selected a rate of λ_f_ = 48 false alarms per hour (0.8 false alarms/min), based on the false alarm rate we computed from a field study using automated detections across 675 h of real field recordings (C. M. Balantic, T. M. Donovan, *unpublished data*).

**Figure 3:**
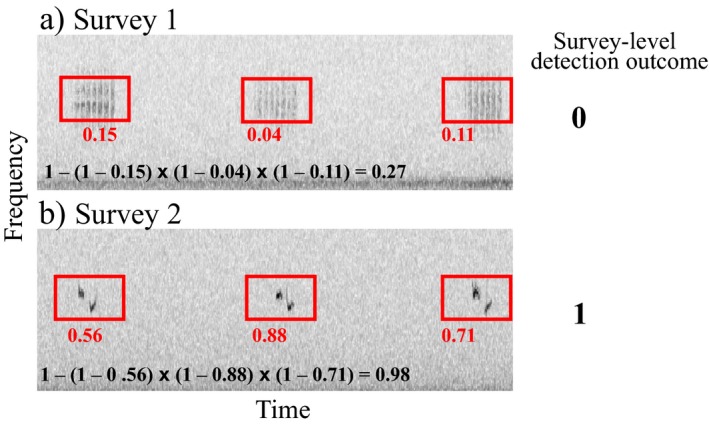
Event‐level detections (red boxes) and their associated target signal probabilities can be aggregated into survey‐level detection outcomes for an encounter history by multiplying together 1 minus the event‐level probabilities within each survey, and then 1 minus this outcome to yield the probability of at least one target signal within the survey (bold black text). (a) Survey 1: If the result does not exceed a user‐defined threshold, such as 0.95, a 0 is assigned at the survey level. (b) Survey 2: If the result does exceed a user‐defined threshold, such as 0.95, the survey is assigned a 1.

Finally, we simulated the production of event‐level detections in each recording within an automated acoustic monitoring framework. Automated detection algorithms, also known as classifiers, may be constructed to produce the probability that an event‐level detection is truly a signal from the target species. For example, in Fig. 3, to each event‐level detection, a trained statistical learning classifier has assigned a probability that the event is a signal from the target species. Hereafter, we refer to this attribute as the “target signal probability” of any event‐level detection. We simulated two alternative classifiers (good and bad), each defined by a mixture of two beta distributions. The good classifier was likely to assign high target signal probabilities to true target signals (which are produced by λ_c_) and low target signal probabilities to false alarms (which are produced by λ_f_; shape parameter α = 4, shape parameter β = 1 for target signals; α = 1, β = 4 for false alarms). The bad classifier was represented by a beta distribution with α = β = 3 for both target signals and false alarms, yielding average target signal probabilities of 0.5 across all detections (Fig. 2c). Table 1 provides an example of event‐level detections with target signal probabilities assigned by good and bad classifiers.

**Table 1:** Illustration of the detection portion of the simulation

Minute	Sound type	Target signal probability	
		Good classifier	Bad classifier
1	false alarm	0.31	0.72
1	false alarm	0.27	0.67
2	target signal	0.80	0.52
2	false alarm	0.04	0.29
2	target signal	0.75	0.46
3	target signal	0.60	0.71
4	target signal	0.87	0.61
4	false alarm	0.07	0.36
5	target signal	0.88	0.35
5	false alarm	0.27	0.09
Mean	false alarm	0.19	0.43
Mean	target signal	0.78	0.53

*Notes*

Event‐level detections can be target signals or false alarms, generated according to λ_c_ and λ_f,_ respectively. For each event‐level detection, a classifier assigns a probability that the detection is actually a target signal. The good classifier typically assigns higher target signal probabilities to actual target signals and lower probabilities to false alarms. The bad classifier makes no such distinction. Both classifiers randomly sample probabilities from their respective beta distributions visualized in Fig. 2c.

In summary, the simulated acoustic environment consisted of four underlying species occurrence dynamics cases, each with two levels of calling production. All eight of these scenarios had the same underlying false alarm rate. Finally, we simulated the automated detection process with two types of classifiers, good and bad, which output the target signal probability associated with each event‐level detection. The Objective 1 simulations thus produced 16 scenarios for subsequent evaluation.

### Encounter history aggregation

To analyze the 16 scenarios produced by Objective 1 under the Miller model occupancy framework, we collapsed event‐level detections into encounter histories (Fig. 2d). Using a capture–recapture framework (sensu Otis et al. 1978), target signal probabilities associated with event‐level detections were aggregated to produce the overall probability that at least one target signal had been detected within a particular survey period, which yields the survey‐level detection. We take care not to conflate the occupancy term “survey” with an individual audio recording: multiple audio recordings might be amassed to collectively constitute the survey based on a chosen unit of survey closure, which is informed by research goals and life history of the target species. To demonstrate, imagine an occupancy survey closure period defined as all of the audio recordings taken in a single day (in our simulation, five minutes of recordings per day are combined into a single survey). Suppose that on the first day (survey 1) three events are detected by the classifier, with target signal probabilities of 0.15, 0.04, and 0.11 (Fig. 3a). In this case, we aggregate the probabilities as (1 − 0.15) × (1 − 0.04) × (1 − 0.11), which gives the probability that *all* detected events are false alarms, 0.73. Next, 1 − 0.73 gives 0.27, the probability that *any* of these detected events is truly a signal from the target. Applying a user‐selected survey‐level threshold of 0.95, we log a 0 in the encounter history for this survey and presume that the species was not detected unless we are 95% sure that we captured at least one target signal. In the next day's survey (Fig. 3b), three events are detected by the automated algorithm, with target signal probabilities of 0.56, 0.88, and 0.71. The probability that all detected events are false alarms is (1 − 0.56) × (1 − 0.88) × (1 − 0.71), resulting in a probability of ~0.015 that all events are false alarms, and probability of ~0.98 that at least one true target signal has been captured by the automated system. Applying the same 0.95 threshold, we log a 1 for this survey to reflect that the target species has been detected in the uncertain state (that is to say, it has been detected automatically and without manual verification). Taken together, the two surveys in this example return an encounter history of 01.

To generate encounter histories for each of the 16 scenarios from Objective 1, we examined eight alternative scenarios defined by three factors: (1) the survey‐level detection threshold, (2) the aggregation period, and (3) the percentage of manually confirmed surveys, which comprise state 2 of the Miller model (Fig. 2d). First, we investigated two survey‐level detection thresholds: 0.80 and 0.95 (i.e., we were 80% or 95% certain that at least one target signal was detected during the survey period). Second, we examined two aggregation options, in which recordings were lumped into a single survey based on a desired unit of closure. We used survey aggregation periods of either one day or three days across each 30‐d monitoring period. Thus, over a 30‐d season, a single season encounter history for the 1‐d aggregation period would yield a string of 30 surveys, represented by a 0, 1, or 2 (with a total of 60 surveys over two seasons). A single season encounter history for the 3‐d aggregation period would produce a string of 30‐d season/3‐d aggregation period = 10 surveys (20 surveys total over two seasons). Last, we examined two scenarios for the total proportion of surveys that were confirmed a posteriori to serve as the “certain” state (state 2) of the Miller model: 0.025 or 0.05. In other words, we randomly assigned 2.5% or 5% of surveys to be manually confirmed to produce a certain state. For practical purposes, with 100 sites across two 30‐d seasons, at a rate of 5 min of recording per site per day, this would equate to manual verification of event‐level detections from 12.5 h (2.5%) or 25 h (5%) worth of recordings, regardless of the *N*‐day survey aggregation period used.

In summary, the 16 acoustic scenarios generated from Objective 1 were each subjected to 8 alternative scenarios for developing encounter histories, resulting in 16 × 8 = 128 total scenarios to be analyzed with the Miller model in Objective 3 (Appendix S1). To summarize the outcome of the simulation, we calculated survey‐level true and false positive rates based on the survey window aggregation length, classifier type, survey‐level detection threshold, and species calling rate. The survey‐level true positive rate indicated the proportion of surveys in the encounter history where the species was present and detected (with rates closest to 1 most desirable). The survey‐level false positive rate signified the proportion of sites where the species was absent but mistakenly detected (with rates closest to 0 most desirable) (note that this *survey*‐level false positive rate should not be confused with the *event*‐level false alarm rate).

### State parameter estimates and bias under different scenarios

The 128 scenarios were simulated 500 times each (64,000 replicates total). To generate parameter estimates for occupancy, colonization, and extinction, we fit intercept models for each of the 64,000 replicates using RPresence V.12.10 (Fig. 2e; Hines 2018). All simulation models were fit using informed initial parameter values to aid convergence to a global minimum in the negative log‐likelihood, because preliminary testing showed that results were sensitive to starting values; we used our known, simulated parameter values as initial starting values. We compared these state parameter estimates to the simulated dynamics values to compute raw bias, as well as the mean and standard deviation across each scenario's 500 replicates (Fig. 2f). Although we focused on the outcomes of the state parameter estimates, we also computed estimate bias for the detection parameters, *p*_11_, *p*_10_, and *b*. We recorded model convergence rates for all scenarios.

## Results

### Simulation of occurrence dynamics, species calling production, and detection process

**Figure 4:**
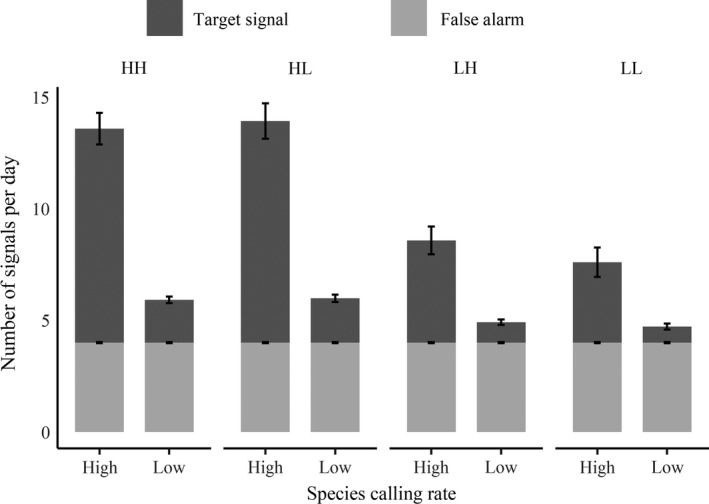
The average daily number of target signals captured by the automated system (dark gray), vs. the average daily number of false alarms captured by the automated system (light gray), across all sites, under both low (20 calls/h) and high (100 calls/h) species‐calling‐production scenarios, and under all four underlying dynamics scenarios (Fig. 2a): high‐occurrence–high‐turnover (HH), high‐occurrence–low‐turnover (HL), low‐occurrence–high‐turnover (LH), and low‐occurrence–low‐turnover (LL).

For the four occurrence‐turnover states and two sound production rates, we summarized daily available sound production averaged across occupied and unoccupied sites in Fig. 4. The total number of species target signals is contingent on occupancy status, given five minutes of recording daily: low occurrence rates naturally produce a lower number of target signals available for automated capture across sites. Meanwhile, the average number of available false alarms is the same regardless of occupancy status and species sound production rate. The good classifier assigned an average target signal probability of 0.80 ± 0.002 (mean ± SD) for target signals and 0.20 ±0.001 for false alarms. The bad classifier assigned an average target signal probability of 0.50 ± 0.003 for target signals and 0.50 ± 0.001 for false alarms.

### Encounter history aggregation

**Figure 5:**
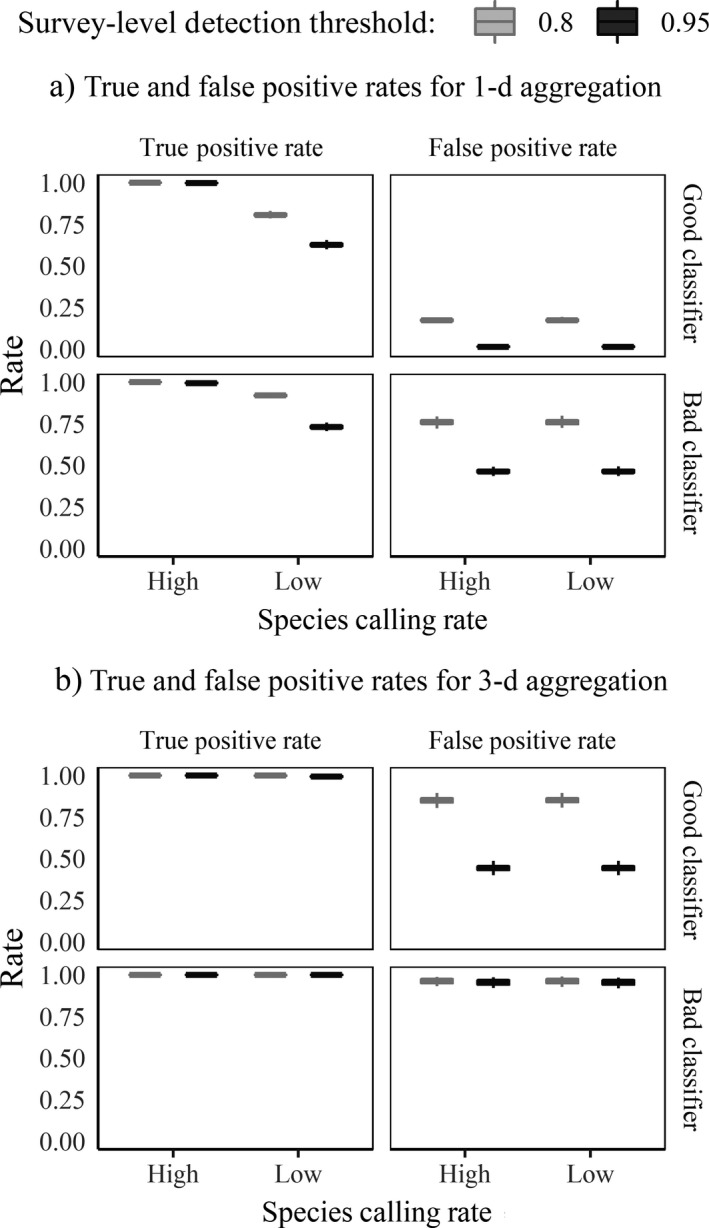
Box plots of survey‐level true positive and false positive rates for (a) 1‐d aggregation and (b) 3‐d aggregation by classifier type, survey‐level detection threshold, and species calling rate (*x*‐axis). Box plots appear as flat, heavy lines with small error bars due to very small variability in the data.

To create encounter histories from the signals produced in Objective 1, recall that we investigated eight alternative scenarios (factors are aggregation day length [1 vs. 3], survey‐level detection threshold [0.8 vs. 0.95], and human‐verified confirmation level [2.5% vs. 5%]). Survey‐level true and false positive rates produced by the automated method differed based on aggregation length, survey‐level detection threshold, and classifier (Fig. 5). Overall, the confirmation levels we chose did not yield any appreciable difference in true and false positive rates. However, rates varied substantially depending on species calling rate, aggregation level, detection threshold, and classifier performance.

For 1‐d aggregation (Fig. 5a), the 0.95 survey‐level detection threshold produced encounter histories with lower underlying true positive rates than those created by the 0.80 threshold, particularly when a good classifier is used against a low call rate (Fig. 5a; upper left panel). It is logical to expect inferior true positive rates for the 0.95 threshold and the good classifier if there are few target signals within a survey period: these conditions foster a higher overall standard that surveys must meet before meriting a score of 1. As a result of this high standard, for both species calling rates, false positive rates for 1‐d aggregation approached zero when using the 0.95 threshold and good classifier; the rate rose to 0.17 when using the 0.80 threshold and good classifier (Fig. 5a; upper right panel). The differences caused by higher and lower standard detection systems illustrate the trade‐off inherent in striving for a high site‐level true positive rate while keeping false positives to a minimum. Overall, the bad classifier generated much higher false positive rates, ranging from 0.46 (0.95 threshold) to 0.75 (0.80 threshold; Fig. 5a; lower right panel).

The trade‐off between a high true positive rate and a low false positive rate is magnified in the 3‐d aggregation scenarios (Fig. 5b). Both survey‐level detection thresholds and both classifiers generated encounter histories with true positive rates of 1 or nearly so. For false positive rates, however, the good classifier had false positive rates as low as 0.44 using the stricter detection threshold (0.95) and false positive rates as high as 0.85 using the lenient threshold (0.8; Fig. 5b; upper right panel). For the bad classifier, false positive rates were near 1 for all encounter history scenarios (Fig. 5b; lower right panel). In summary, although 3‐d aggregation generated encounter history scenarios with higher underlying survey‐level true positive rates overall, these encounter history scenarios also carried higher underlying false positive rates. Meanwhile, 1‐d aggregation produced encounter history scenarios with lower overall true positive rates, but also much lower false positive rates.

### State parameter estimates and bias under different scenarios

**Figure 6:**
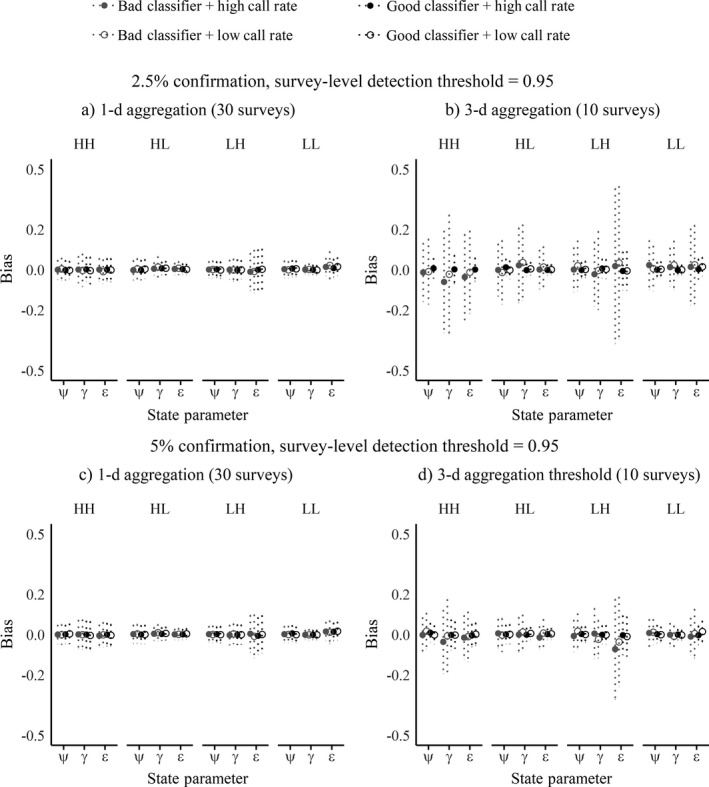
Summary of state parameter estimate bias across occurrence dynamics, species call rates, classifier performance, aggregation frames, and confirmation percentages, at the 0.95 survey‐level detection threshold. Circles indicate the mean bias, with dotted vertical bars showing standard deviations. Open circles denote scenarios with a low call rate. Closed circles denote a high call rate. Gray circles denote the bad classifier, and black circles denote the good classifier. Note that the *y*‐axis ranges from −0.5 to 0.5. (a) 1‐d aggregation, 2.5% confirmation, survey‐level detection threshold = 0.95. (b) 3‐d aggregation, 2.5% confirmation, survey‐level detection threshold = 0.95. (c) 1‐d aggregation, 5% confirmation, survey‐level detection threshold = 0.95. (d) 3‐d aggregation, 5% confirmation, survey‐level detection threshold = 0.95.

Summarized across all dynamics and calling scenarios, encounter histories generated with the 1‐d survey aggregation period generally produced the least biased estimates across the three state parameters, with bias values closest to zero being most desirable. We focus on results for the 0.95 survey‐level detection threshold (Fig. 6), while results for the 0.80 threshold are contained in Appendix S2. The superiority of the smaller aggregation period held true across both survey‐level detection thresholds, both the good and bad classifiers, and both proportions of a posteriori survey confirmation. Under 1‐d aggregation, neither confirmation level nor survey‐level detection threshold made an appreciable difference in the bias estimates (Fig. 6a, c; Appendix S2: Fig. S1 a,c). Under 3‐d aggregation, the higher confirmation level (5%) reduced both the bias and variation in bias (compare Fig. 6b–d; Appendix S2: Fig. S1b–d). Generally, when a good classifier was used, bias was comparable across all Fig. 6 scenario panels. Thus, although 1‐d aggregation outperformed 3‐d aggregation overall, 3‐d aggregation is competitive with increasing survey‐level detection thresholds and/or confirmation levels, especially if the classifier is good, demonstrating that longer aggregation windows can retain utility if adequately balanced by higher automated detection standards and higher manual confirmation effort.

**Figure 7:**
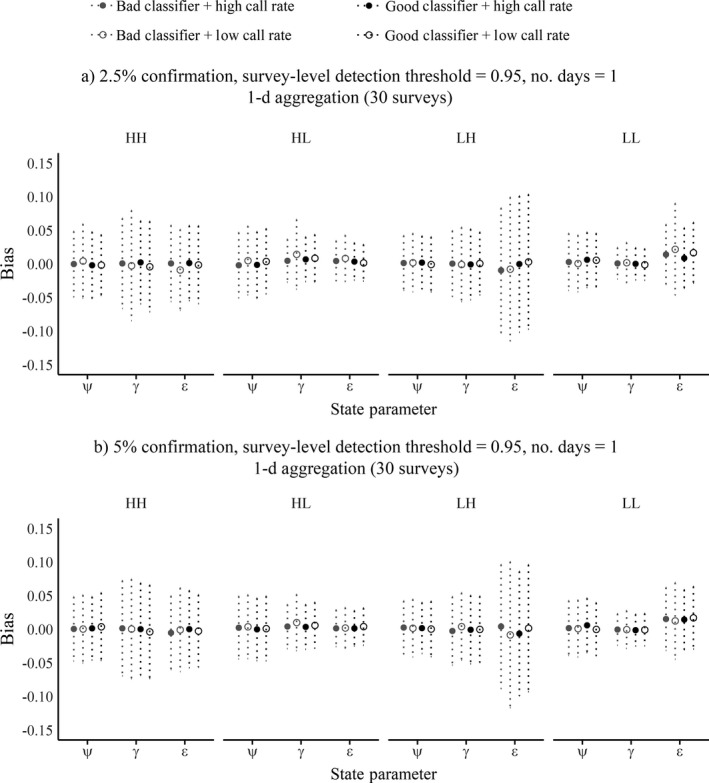
Summary of state parameter estimate bias across different dynamics, call production, classifier performance, and confirmation percentages, for the most conservative survey aggregation circumstances (aggregate days = 1, survey‐level detection threshold = 0.95). Circles indicate the mean bias, with dotted vertical bars showing standard deviations. Note that the *y*‐axis has narrowed to range from −0.15 to 0.15. (a) 1‐d aggregation, 2.5% confirmation, survey‐level detection threshold = 0.95. (b) 1‐d aggregation, 5% confirmation, survey‐level detection threshold = 0.95.

**Figure 8:**
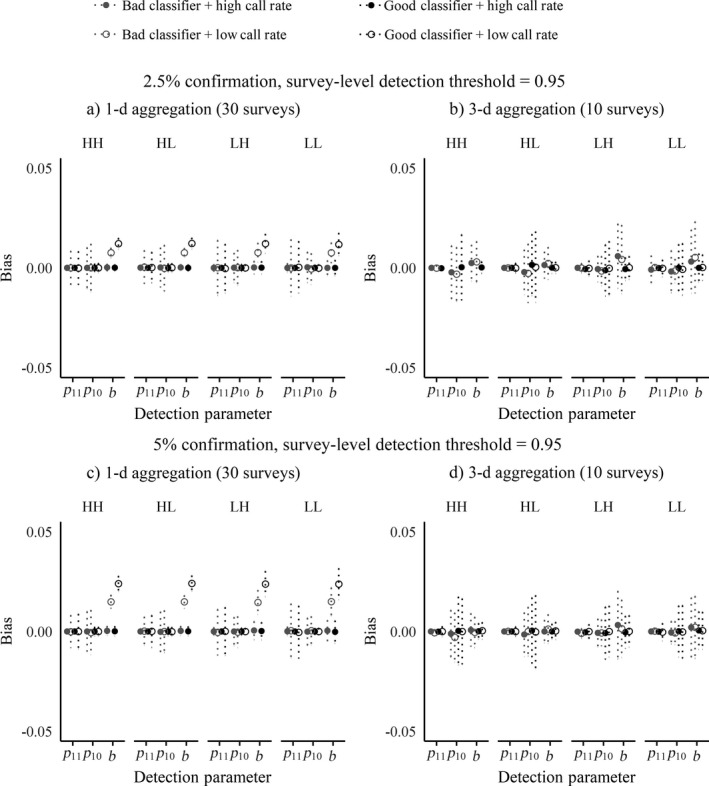
Summary of detection parameter estimate bias across different dynamics, call production, classifier performance, and confirmation percentages, at the 0.95 survey‐level detection threshold. Circles indicate the mean bias, with dotted vertical bars showing standard deviations. Note that the *y*‐axis has narrowed to range from −0.05 to 0.05. The parameter *p* is the probability of detecting a truly present species, where *p*_10_ represents the probability of incorrectly detecting the species at an unoccupied site (survey‐level false positive) and *p*_11_ is the probability of correctly detecting a present species at an occupied site (survey‐level true positive). Certain detections are denoted by the parameter *b*, the probability of detecting a species via automated detection paired with manual verification, given presence. (a) 1‐d aggregation, 2.5% confirmation, survey‐level detection threshold = 0.95. (b) 3‐d aggregation, 2.5% confirmation, survey‐level detection threshold = 0.95. (c) 1‐d aggregation, 5% confirmation, survey‐level detection threshold = 0.95. (d) 3‐d aggregation, 5% confirmation, survey‐level detection threshold = 0.95. HH, high initial occurrence with high turnover; HL, high initial occurrence with low turnover; LH, low initial occurrence with high turnover; LL, low initial occurrence with low turnover.

Under most scenarios, state parameter estimates tended to have wide ranges in variation. Zooming in on the most conservative encounter history aggregation conditions (1‐d aggregation, 0.95 survey‐level detection threshold, Fig. 7), mean estimates fell within 3% of simulated truth, though with deviation out to 10% in both directions for some parameters. The lower confirmation level (Fig. 7a) was generally competitive with the higher confirmation level (Fig. 7b). The high‐occurrence–low‐turnover (HL) scenario had the least biased and most precise estimates across all three parameters under all scenarios and both confirmation levels, and the low‐occurrence–low‐turnover (LL) scenario would have approached this level of precision if not for the tendency to overestimate the extinction parameter (ε). The higher turnover scenarios (HH, LH) generally produced more variation in the bias. The influence of species calling rate was minimal overall. Estimates for the detection parameters *p*_11_, *p*_10_, and *b* were generally much less biased and more precise than the state parameter estimates, with mean biases and standard deviations all falling well within 3% of simulated truth (Fig. 8; Appendix S2 Fig. S2).

The Miller model convergence rate across all scenarios was 59%. Only replicates that converged were included in the Figs. 6 and 7 results. Convergence rates generally mirrored the bias results. The number of aggregation days had the clearest impact on convergence: 1‐d aggregation had an 84% convergence rate, while 3‐d aggregation only converged 34% of the time. Classifier type also affected convergence, with the good classifier (68% convergence rate) outperforming the bad classifier (51% convergence rate). Survey detection level also affected convergence rates: models that used a 0.95 threshold converged 66% of the time, whereas models with a 0.80 threshold converged at a rate of 53%. The impact of confirmation level was minimal (2.5% confirmation: 57% converged, 5% confirmation: 61% converged). Convergence rates also differed based on the underlying state dynamics, with the high turnover scenarios (HH, LH) converging at greater rates overall (HH: 67%, HL: 57%, LH: 64%, and LL: 50%). The high calling rate (55%) converged substantially less frequently than the low calling rate (64%).

## Discussion

The capacity to understand and predict long‐term, large‐scale, species occurrence dynamics is critical against a backdrop of climate change and rapidly shifting land uses (Nichols et al. 2015). Although automated acoustic monitoring provides a means for cost‐effective and efficient collection of species occurrence data, minimal guidance exists for translating enormous streams of raw audio data into dynamic occurrence models that provide actual utility for wildlife researchers and land managers. We introduced a novel method for aggregating detected events into encounter histories for use in dynamic models meant to capture changes in occurrence patterns over time. When automated detection algorithms are constructed to yield the probability that a detected event is produced by a target signal, these event‐level probabilities may be aggregated within a capture–recapture framework to provide the probability that *any* detected event within a survey period is a sound from the target species (Otis et al. 1978). We believe this work is the first to unify the concepts of automated acoustic data collection with analysis for mistake‐sensitive dynamic occupancy modeling, although single‐season approaches have been implemented (Kalan et al. 2015, Cerqueira and Aide 2016, Chambert et al. 2017). Where classifier‐assigned target signal probabilities are not available, Chambert et al. (2017) offer a method contingent on the total abundance of event‐level detections. Other alternatives include automated target event detection followed by manual cleaning (Kalan et al. 2015, Cerqueira and Aide 2016), or deploying machine learning approaches to identify and remove false positives automatically. In contrast to removing false positives manually or automatically, the method we describe here takes full advantage of the information provided by target signal probabilities associated with each detected event.

To explore the utility of the Miller model framework for dynamic occupancy models constructed from automated acoustic monitoring data, we investigated our probability aggregation method in 128 scenarios that spanned a range of occurrence dynamics, species sound production rates, classifier performances, and encounter history aggregation. Our results demonstrate that the Miller model was able to produce state parameter estimates within an average of 3% of simulated truth estimates for occurrence, colonization, and extinction for all latent conditions (dynamics and sound production) and all observation conditions (good vs. bad classifier), given specific encounter history aggregation choices. We also applied our probability aggregation method to the false positive, ignorant dynamic model (MacKenzie et al. 2003) but found that it was generally not competitive with the Miller model. In a 100‐repetition test, the model of MacKenzie et al. 2003 tended to overestimate initial occurrence and underestimate extinction rates (Appendix S3), suggesting that the Miller model is the stronger choice for automated acoustic monitoring.

In our simulation, narrow frames of survey aggregation produced the least biased parameter estimates. These shorter survey aggregation periods, in turn, produce a larger number of surveys. Single‐day aggregation outperformed 3‐d aggregation because longer aggregation periods are more likely to result in “uncertain” survey‐level false positives in the encounter history, particularly if the survey‐level detection threshold is not high enough. Longer aggregation periods lead to a greater number of detected events. If a species is absent from a site, even if a good classifier is used, the target signal probabilities assigned to false alarms may not be low enough to overcome the effects of many probabilities ultimately being multiplied together. The (multiplied) product of too many probabilities may be an unacceptably high number of survey‐level false positives within the probability aggregation scheme (as in Fig. 5b). Although the 3‐d aggregation period slightly outperforms 1‐d aggregation on the survey‐level true positive rate, the accompanying bloated false positive rates are too high to overcome without bias when fitting the dynamic occupancy model. Thus, smaller windows of probability aggregation may perform better in general, though the narrowness of this aggregation window must be balanced against practical considerations for the period of survey closure for a target species.

These general results bode well for an automated acoustic monitoring program. Automated acoustic monitoring, like camera trapping and other remote automated methods, boasts a unique position in the occupancy modeling realm, where many traditional study methods tend to be “survey poor” (MacKenzie and Royle 2005). In contrast, automated acoustic monitoring provides the opportunity to be “survey rich” if audio recordings occur regularly over an extended time, a benefit of the flexibility around gathering audio recordings into distinct survey periods. Since false positives can inflate the number of recommended surveys (Clement 2016), the opportunity to be survey rich is useful for a monitoring methodology where false positives are prevalent. The opportunity to be survey rich is similarly useful for mitigating survey‐level false negatives. Accounting for detection probability is likely even more important when underlying species occupancy is low, though the relative ease of repeated long‐term surveying offered by ARUs may ameliorate false negatives and improve the precision of occupancy estimates (Kalan et al. 2015). Additionally, although in this simulation we used a human survey confirmation method focused strictly on cleaning out false positives (wherein survey confirmation was conducted entirely based on manual verification of automatically detected events), false negatives may be more directly addressed by extending the idea of “confirmed surveys” to include intensive human manual annotation of recordings from the entire survey period (in contrast to the less labor‐intensive, false‐positive‐focused confirmation method of simply verifying automatically detected events). In real field applications, survey‐level false negatives in acoustic monitoring may also be mitigated via temporally adaptive sampling methodology, which explicitly strives to avoid false negatives by recording when target species are likely to be acoustically active.

Unsurprisingly, a higher quality classifier will better serve an acoustic monitoring program than a poor classifier. In our experiment, the good classifier was typically able to provide minimally biased results even when coping with a long aggregation period (Fig. 6d), or low survey‐level detection thresholds provided that the aggregation period was short enough (Appendix S2: Fig. S1a,c), while the bad classifier was often less robust under these conditions. For the good classifier, as long as the aggregation period was short, the confirmation levels we examined made little difference. While we expect that no research team would intentionally deploy a bad classifier, the performance of an automated detection system during the testing phase can be markedly different from its performance on new audio data, which can introduce emergent challenges such as seasonal changes to the soundscape, turnover of individual animals that contributed training data to the automated detection system, and cultural drift of vocalization behavior over time (Williams et al. 2013). Researchers should take caution in the deployment of automated detection systems that have not been thoroughly field tested (Russo and Voigt 2016). To moderate the impacts of these potential changes on classifier performance over time, a monitoring team may opt for higher confirmation levels, or might choose to place their automated detection and classification models in a Bayesian framework, updating them regularly at intervals appropriate for the target species and study landscape. Additionally, if target species issue multiple types of call or song signals, multiple detectors and classifiers can be used to scan audio files; our method can easily incorporate such cases.

Compared with short‐term ecological monitoring studies, long‐term studies have a disproportionately large impact on scientific knowledge and policy (Hughes et al. 2017), and are more likely to have conservation and land management utility compared with static, single‐season approaches (Dugger et al. 2015). Automated acoustic monitoring may support this type of research by translating audio data streams into dynamic occupancy models (sensu MacKenzie et al. 2003 or Miller et al. 2013). We are unaware of any real field examples of dynamic occupancy models constructed from automated acoustic monitoring data, although several examples exist for single‐season models (Furnas and Callas 2015, Kalan et al. 2015, Cerqueira and Aide 2016). If monitoring were to continue in these cases, the associated research programs would be well positioned to conduct long‐term, landscape‐scale, ecological monitoring with acoustic data. Such frameworks may support adaptive management paradigms across landscapes, amid climate change and land use change. Adaptive management uses learning over time to inform future management decisions (Williams et al. 2009), but it can be challenging to implement. The U.S. Fish and Wildlife Agency's adaptive management program for American waterfowl populations provides one example of a systematic implementation. This approach employs a mixture of management objectives, monitoring, and model prediction under alternative management scenarios to achieve optimal management decisions by way of reductions in uncertainty around population responses to land management over time (Nichols et al. 2007, Johnson et al. 2015). Though no examples yet exist for adaptive management using dynamic occupancy models derived from acoustic monitoring data, emerging tools for codifying models and tracking research and management objectives through time (e.g., the R package AMModels; Donovan and Katz 2018) make this an imminent possibility.

In real field settings, researchers aiming to conduct dynamic occupancy modeling will likely confront logistical challenges such as the replacement of equipment, technological improvements, and refinement of acoustic sampling techniques through time that could compromise data consistency across multiple seasons if not adequately addressed (Shonfield and Bayne 2017). Additionally, dynamic occupancy model frameworks require larger site sample sizes to provide reliable estimates of the additional parameters in dynamic models, a reality with which acoustic monitoring is compatible given increasing emergence of highly cost‐efficient recording units (e.g. Solo, ~US$190 per unit [Whytock and Christie 2017]; AudioMoth (Open Acoustic Devices, University of Southampton, Southampton, UK and Oxford University, Oxford, UK), US$43 per unit [Hill et al. 2018]). However, the utility of long‐term, large‐scale, acoustic monitoring will be undercut without a means for moving from raw acoustic data to population models from which inference may be gained. Generation of occupancy model encounter histories from large data streams is a salient challenge in automated acoustic monitoring, and likely to become more so with increasing adoption of automated acoustic monitoring methods.

In this work, we conducted all simulations using the acoustic monitoring data management framework described in the AMMonitor package (C. M. Balantic, J. E. Katz, T. M. Donovan, *unpublished manuscript*), which contains functions to support machine learning assignment of target signal probability values to automatically detected events, in addition to functions supporting the aggregation of automated detection data into encounter histories for use in dynamic occupancy models.
