# Supplementary material for: Dynamic wildlife occupancy models using automated acoustic monitoring data
Source: Ecol Appl. 2019 Feb 27;29(3):e01854. doi: 10.1002/eap.1854 (PMC6852693; doi:10.1002/eap.1854)
Supplement: Supplementary file 1 [file EAP-29-na-s001.pdf]

Supporting Information. Balantic, C. M. and T. M. Donovan. 2019. Dynamic wildlife occupancy models using automated acoustic monitoring data. *Ecological Applications.*

Appendix S1

Table S1. Exhaustive table of 128 scenarios used in the simulation.

Scenario	Dynamics	Sound Production	Automated Detection	Survey-level Detection Threshold	Survey Aggregation Length	Confirmation Effort Level
1	High Occurrence, High Turnover	High	Bad	0.95	1-day	0.025
2	High Occurrence, High Turnover	High	Bad	0.95	1-day	0.05
3	High Occurrence, High Turnover	High	Bad	0.95	3-day	0.025
4	High Occurrence, High Turnover	High	Bad	0.95	3-day	0.05
5	High Occurrence, High Turnover	High	Bad	0.8	1-day	0.025
6	High Occurrence, High Turnover	High	Bad	0.8	1-day	0.05
7	High Occurrence, High Turnover	High	Bad	0.8	3-day	0.025
8	High Occurrence, High Turnover	High	Bad	0.8	3-day	0.05
9	High Occurrence, High Turnover	High	Good	0.95	1-day	0.025
10	High Occurrence, High Turnover	High	Good	0.95	1-day	0.05
11	High Occurrence, High Turnover	High	Good	0.95	3-day	0.025
12	High Occurrence, High Turnover	High	Good	0.95	3-day	0.05
13	High Occurrence, High Turnover	High	Good	0.8	1-day	0.025
14	High Occurrence, High Turnover	High	Good	0.8	1-day	0.05
15	High Occurrence, High Turnover	High	Good	0.8	3-day	0.025
16	High Occurrence, High Turnover	High	Good	0.8	3-day	0.05
17	High Occurrence, High Turnover	Low	Bad	0.95	1-day	0.025
18	High Occurrence, High Turnover	Low	Bad	0.95	1-day	0.05
19	High Occurrence, High Turnover	Low	Bad	0.95	3-day	0.025
20	High Occurrence, High Turnover	Low	Bad	0.95	3-day	0.05
21	High Occurrence, High Turnover	Low	Bad	0.8	1-day	0.025
22	High Occurrence, High Turnover	Low	Bad	0.8	1-day	0.05
23	High Occurrence, High Turnover	Low	Bad	0.8	3-day	0.025
24	High Occurrence, High Turnover	Low	Bad	0.8	3-day	0.05
25	High Occurrence, High Turnover	Low	Good	0.95	1-day	0.025
26	High Occurrence, High Turnover	Low	Good	0.95	1-day	0.05
27	High Occurrence, High Turnover	Low	Good	0.95	3-day	0.025
28	High Occurrence, High Turnover	Low	Good	0.95	3-day	0.05
29	High Occurrence, High Turnover	Low	Good	0.8	1-day	0.025
30	High Occurrence, High Turnover	Low	Good	0.8	1-day	0.05
31	High Occurrence, High Turnover	Low	Good	0.8	3-day	0.025

32	High Occurrence, High Turnover	Low	Good	0.8	3-day	0.05
33	High Occurrence, Low Turnover	High	Bad	0.95	1-day	0.025
34	High Occurrence, Low Turnover	High	Bad	0.95	1-day	0.05
35	High Occurrence, Low Turnover	High	Bad	0.95	3-day	0.025
36	High Occurrence, Low Turnover	High	Bad	0.95	3-day	0.05
37	High Occurrence, Low Turnover	High	Bad	0.8	1-day	0.025
38	High Occurrence, Low Turnover	High	Bad	0.8	1-day	0.05
39	High Occurrence, Low Turnover	High	Bad	0.8	3-day	0.025
40	High Occurrence, Low Turnover	High	Bad	0.8	3-day	0.05
41	High Occurrence, Low Turnover	High	Good	0.95	1-day	0.025
42	High Occurrence, Low Turnover	High	Good	0.95	1-day	0.05
43	High Occurrence, Low Turnover	High	Good	0.95	3-day	0.025
44	High Occurrence, Low Turnover	High	Good	0.95	3-day	0.05
45	High Occurrence, Low Turnover	High	Good	0.8	1-day	0.025
46	High Occurrence, Low Turnover	High	Good	0.8	1-day	0.05
47	High Occurrence, Low Turnover	High	Good	0.8	3-day	0.025
48	High Occurrence, Low Turnover	High	Good	0.8	3-day	0.05
49	High Occurrence, Low Turnover	Low	Bad	0.95	1-day	0.025
50	High Occurrence, Low Turnover	Low	Bad	0.95	1-day	0.05
51	High Occurrence, Low Turnover	Low	Bad	0.95	3-day	0.025
52	High Occurrence, Low Turnover	Low	Bad	0.95	3-day	0.05
53	High Occurrence, Low Turnover	Low	Bad	0.8	1-day	0.025
54	High Occurrence, Low Turnover	Low	Bad	0.8	1-day	0.05
55	High Occurrence, Low Turnover	Low	Bad	0.8	3-day	0.025
56	High Occurrence, Low Turnover	Low	Bad	0.8	3-day	0.05
57	High Occurrence, Low Turnover	Low	Good	0.95	1-day	0.025
58	High Occurrence, Low Turnover	Low	Good	0.95	1-day	0.05
59	High Occurrence, Low Turnover	Low	Good	0.95	3-day	0.025
60	High Occurrence, Low Turnover	Low	Good	0.95	3-day	0.05
61	High Occurrence, Low Turnover	Low	Good	0.8	1-day	0.025
62	High Occurrence, Low Turnover	Low	Good	0.8	1-day	0.05
63	High Occurrence, Low Turnover	Low	Good	0.8	3-day	0.025
64	High Occurrence, Low Turnover	Low	Good	0.8	3-day	0.05
65	Low Occurrence, High Turnover	High	Bad	0.95	1-day	0.025
66	Low Occurrence, High Turnover	High	Bad	0.95	1-day	0.05
67	Low Occurrence, High Turnover	High	Bad	0.95	3-day	0.025
68	Low Occurrence, High Turnover	High	Bad	0.95	3-day	0.05
69	Low Occurrence, High Turnover	High	Bad	0.8	1-day	0.025
70	Low Occurrence, High Turnover	High	Bad	0.8	1-day	0.05
71	Low Occurrence, High Turnover	High	Bad	0.8	3-day	0.025
72	Low Occurrence, High Turnover	High	Bad	0.8	3-day	0.05
73	Low Occurrence, High Turnover	High	Good	0.95	1-day	0.025
74	Low Occurrence, High Turnover	High	Good	0.95	1-day	0.05
75	Low Occurrence, High Turnover	High	Good	0.95	3-day	0.025

76	Low Occurrence, High Turnover	High	Good	0.95	3-day	0.05
77	Low Occurrence, High Turnover	High	Good	0.8	1-day	0.025
78	Low Occurrence, High Turnover	High	Good	0.8	1-day	0.05
79	Low Occurrence, High Turnover	High	Good	0.8	3-day	0.025
80	Low Occurrence, High Turnover	High	Good	0.8	3-day	0.05
81	Low Occurrence, High Turnover	Low	Bad	0.95	1-day	0.025
82	Low Occurrence, High Turnover	Low	Bad	0.95	1-day	0.05
83	Low Occurrence, High Turnover	Low	Bad	0.95	3-day	0.025
84	Low Occurrence, High Turnover	Low	Bad	0.95	3-day	0.05
85	Low Occurrence, High Turnover	Low	Bad	0.8	1-day	0.025
86	Low Occurrence, High Turnover	Low	Bad	0.8	1-day	0.05
87	Low Occurrence, High Turnover	Low	Bad	0.8	3-day	0.025
88	Low Occurrence, High Turnover	Low	Bad	0.8	3-day	0.05
89	Low Occurrence, High Turnover	Low	Good	0.95	1-day	0.025
90	Low Occurrence, High Turnover	Low	Good	0.95	1-day	0.05
91	Low Occurrence, High Turnover	Low	Good	0.95	3-day	0.025
92	Low Occurrence, High Turnover	Low	Good	0.95	3-day	0.05
93	Low Occurrence, High Turnover	Low	Good	0.8	1-day	0.025
94	Low Occurrence, High Turnover	Low	Good	0.8	1-day	0.05
95	Low Occurrence, High Turnover	Low	Good	0.8	3-day	0.025
96	Low Occurrence, High Turnover	Low	Good	0.8	3-day	0.05
97	Low Occurrence, Low Turnover	High	Bad	0.95	1-day	0.025
98	Low Occurrence, Low Turnover	High	Bad	0.95	1-day	0.05
99	Low Occurrence, Low Turnover	High	Bad	0.95	3-day	0.025
100	Low Occurrence, Low Turnover	High	Bad	0.95	3-day	0.05
101	Low Occurrence, Low Turnover	High	Bad	0.8	1-day	0.025
102	Low Occurrence, Low Turnover	High	Bad	0.8	1-day	0.05
103	Low Occurrence, Low Turnover	High	Bad	0.8	3-day	0.025
104	Low Occurrence, Low Turnover	High	Bad	0.8	3-day	0.05
105	Low Occurrence, Low Turnover	High	Good	0.95	1-day	0.025
106	Low Occurrence, Low Turnover	High	Good	0.95	1-day	0.05
107	Low Occurrence, Low Turnover	High	Good	0.95	3-day	0.025
108	Low Occurrence, Low Turnover	High	Good	0.95	3-day	0.05
109	Low Occurrence, Low Turnover	High	Good	0.8	1-day	0.025
110	Low Occurrence, Low Turnover	High	Good	0.8	1-day	0.05
111	Low Occurrence, Low Turnover	High	Good	0.8	3-day	0.025
112	Low Occurrence, Low Turnover	High	Good	0.8	3-day	0.05
113	Low Occurrence, Low Turnover	Low	Bad	0.95	1-day	0.025
114	Low Occurrence, Low Turnover	Low	Bad	0.95	1-day	0.05
115	Low Occurrence, Low Turnover	Low	Bad	0.95	3-day	0.025
116	Low Occurrence, Low Turnover	Low	Bad	0.95	3-day	0.05
117	Low Occurrence, Low Turnover	Low	Bad	0.8	1-day	0.025
118	Low Occurrence, Low Turnover	Low	Bad	0.8	1-day	0.05
119	Low Occurrence, Low Turnover	Low	Bad	0.8	3-day	0.025

120	Low Occurrence, Low Turnover	Low	Bad	0.8	3-day	0.05
121	Low Occurrence, Low Turnover	Low	Good	0.95	1-day	0.025
122	Low Occurrence, Low Turnover	Low	Good	0.95	1-day	0.05
123	Low Occurrence, Low Turnover	Low	Good	0.95	3-day	0.025
124	Low Occurrence, Low Turnover	Low	Good	0.95	3-day	0.05
125	Low Occurrence, Low Turnover	Low	Good	0.8	1-day	0.025
126	Low Occurrence, Low Turnover	Low	Good	0.8	1-day	0.05
127	Low Occurrence, Low Turnover	Low	Good	0.8	3-day	0.025
128	Low Occurrence, Low Turnover	Low	Good	0.8	3-day	0.05
