# Supplementary material for: Dynamic wildlife occupancy models using automated acoustic monitoring data
Source: Ecol Appl. 2019 Feb 27;29(3):e01854. doi: 10.1002/eap.1854 (PMC6852693; doi:10.1002/eap.1854)

Supporting Information. Balantic, C. M. and T. M. Donovan. 2019. Dynamic wildlife occupancy models using automated acoustic monitoring data. *Ecological Applications.*

Appendix S2

State and detection parameter estimate bias across occurrence dynamics, species call rates, classifier performance, aggregation frames, and confirmation percentages, at the 0.8 survey-level detection threshold. Circles indicate the mean bias, with dotted vertical bars showing standard deviations. Open circles denote scenarios with a low call rate. Closed circles denote a high call rate. Gray circles denote the bad classifier, and black circles denote the good classifier.

Appendix S2: Figure S1. Summary of state parameter estimate bias at the 0.8 survey-level detection threshold. Note that the y-axis ranges from -0.5 to 0.5.

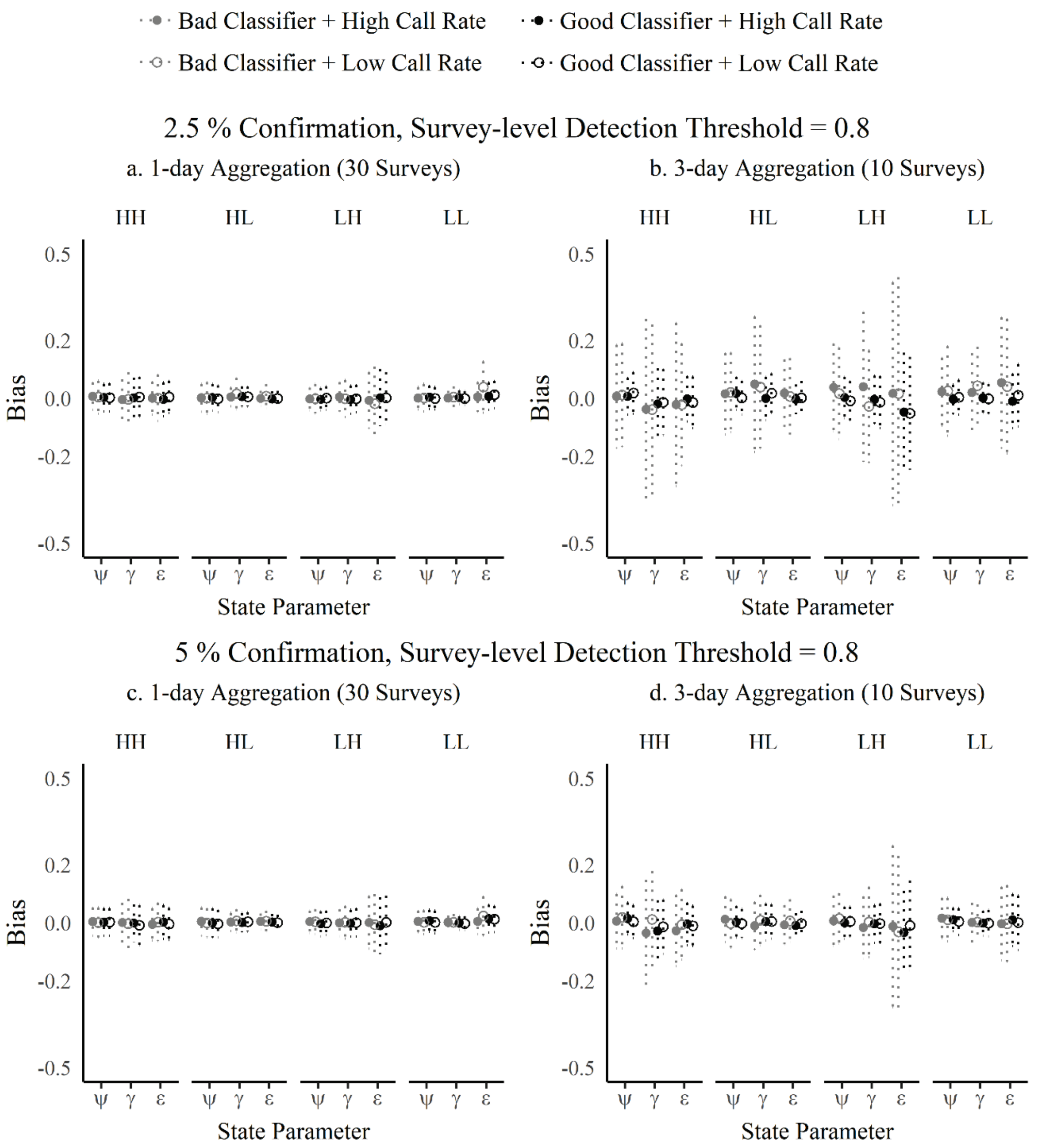

Appendix S2: Figure S2. Summary of detection parameter estimate bias at the 0.8 survey-level detection threshold. Note that the y-axis has narrowed to range from -0.05 to 0.05.

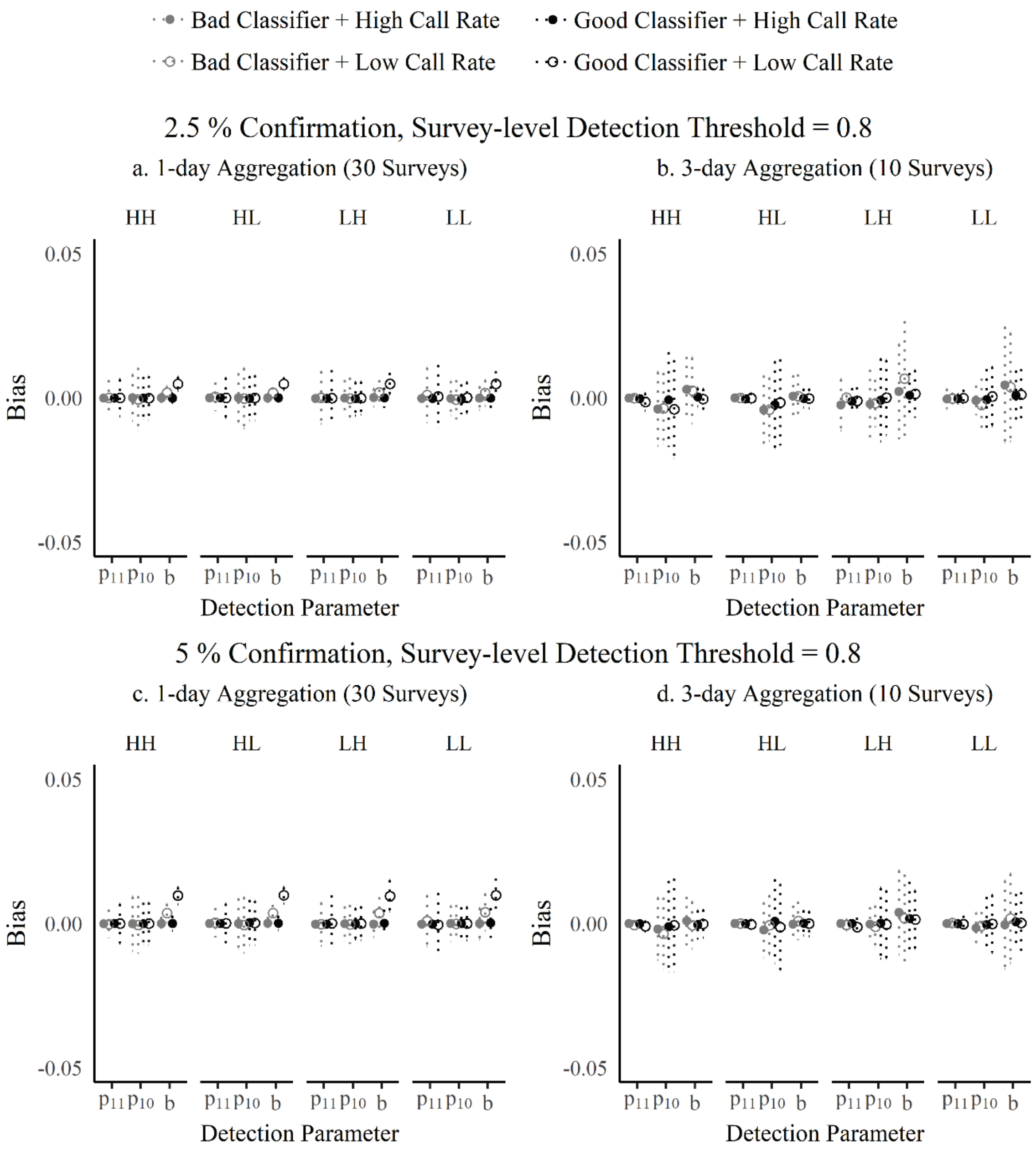

Supplement: Supplementary file 2 [file EAP-29-na-s002.pdf]
