# Supplementary material for: Dynamic wildlife occupancy models using automated acoustic monitoring data
Source: Ecol Appl. 2019 Feb 27;29(3):e01854. doi: 10.1002/eap.1854 (PMC6852693; doi:10.1002/eap.1854)
Supplement: Supplementary file 3 [file EAP-29-na-s003.pdf]

Supporting Information. Balantic, C. M. and T. M. Donovan. 2019. Dynamic wildlife occupancy models using automated acoustic monitoring data. *Ecological Applications.*

Appendix S3

Performance of the classic dynamic occupancy model that ignores false positives (Mackenzie *et al.* 2003) in a 100-replicate experiment. Summary of parameter estimate bias across occurrence dynamics, species call rates, classifier performance, aggregation frames, survey-level detection thresholds, and confirmation percentages. Circles indicate the mean bias, with dotted vertical bars showing standard deviations. Open circles denote scenarios with a low call rate. Closed circles denote a high call rate. Gray circles denote the bad classifier, and black circles denote the good classifier.

Appendix S3: Figure S1. Performance of the classic (false positive-ignorant) dynamic model in a 100-replicate experiment: state parameter results.

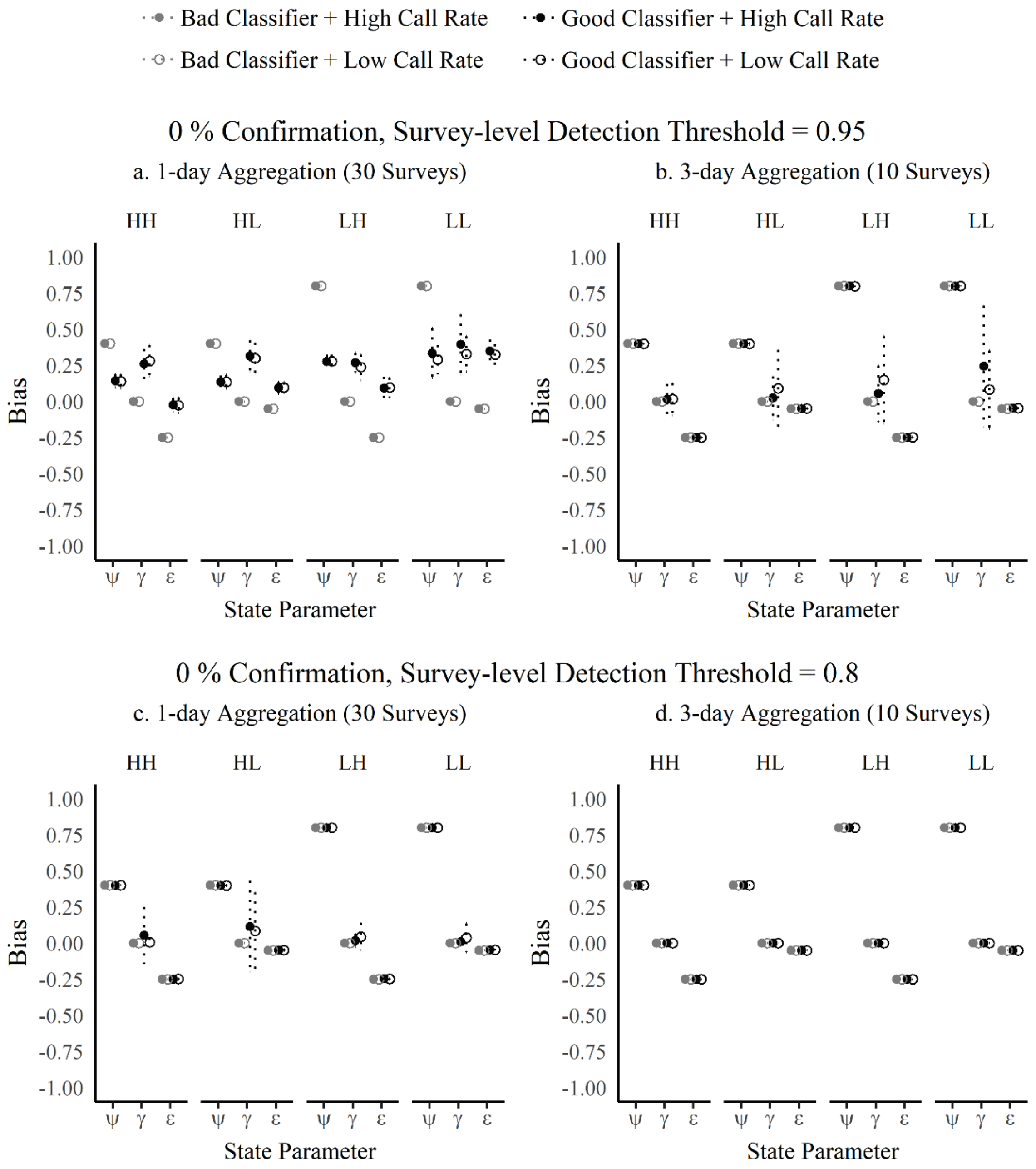

Appendix S3: Figure S2. Performance of the classic (false positive-ignorant) dynamic model in a 100-replicate experiment: detection parameter results.

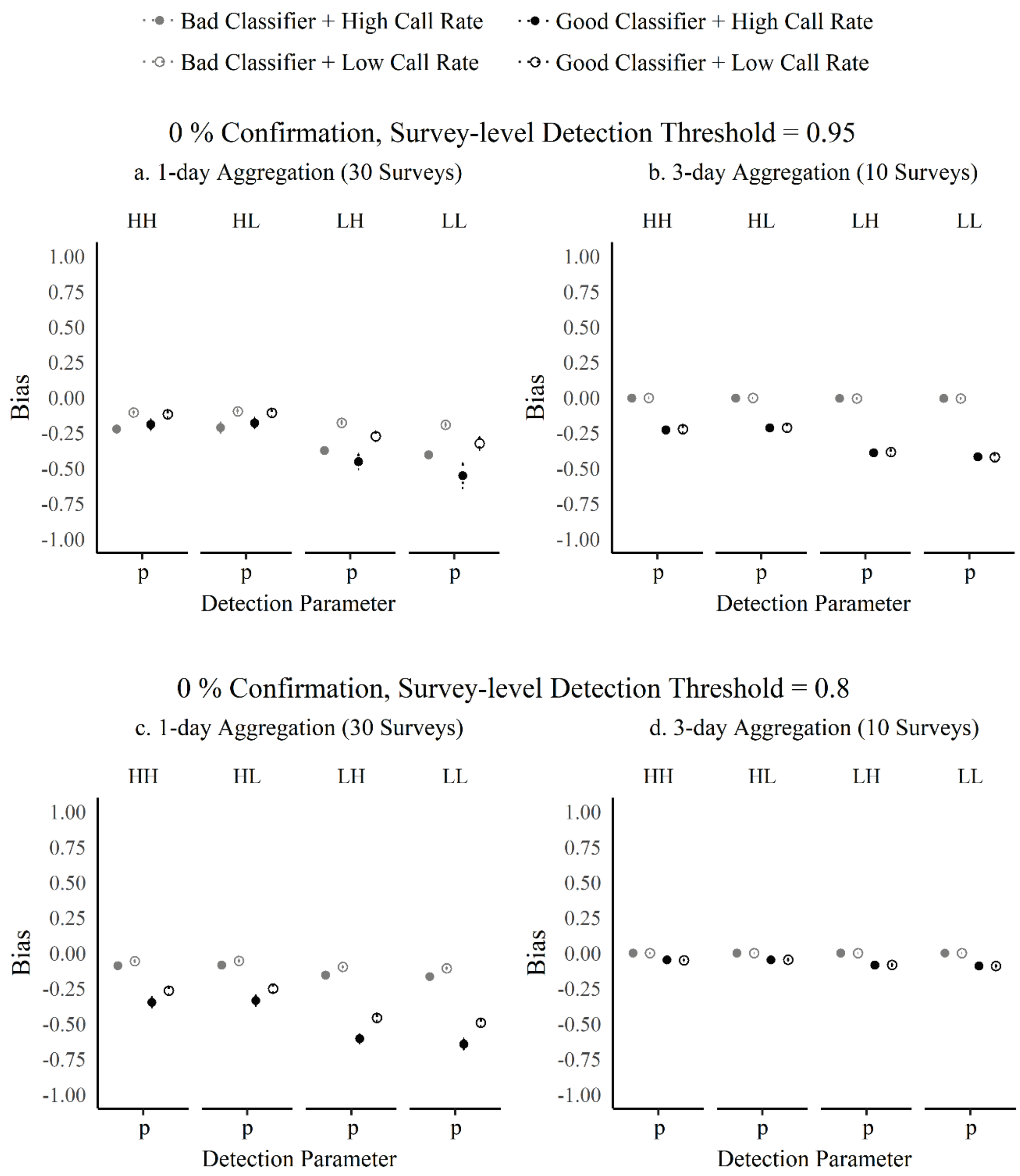

Literature Cited

MacKenzie, D. I., J. D. Nichols, J. E. Hines, M. G. Knutson, and A.B. Franklin. 2003. Estimating site occupancy, colonization, and local extinction when a species is detected imperfectly. Ecology 84:2200–2207.
