# Supplementary material for: Dynamic wildlife occupancy models using automated acoustic monitoring data
Source: Ecol Appl. 2019 Feb 27;29(3):e01854. doi: 10.1002/eap.1854 (PMC6852693; doi:10.1002/eap.1854)
Supplement: Supplementary file 5 [file EAP-29-na-s005.pdf]

Balantic, C. M. and T. M. Donovan. Dynamic wildlife occupancy models using automated acoustic monitoring data. *Ecological Applications.* 2019.

Data S1

Code and data to reproduce the simulations described in "Dynamic wildlife occupancy models using automated acoustic monitoring data"

Authors of the material provided in DataS1.zip

Cathleen Balantic
Vermont Cooperative Fish and Wildlife Research Unit
302 Aiken Center, 81 Carrigan Drive, University of Vermont, Burlington, VT 05405, USA
cathleen.balantic@uvm.edu

Therese Donovan
U.S. Geological Survey, Vermont Cooperative Fish and Wildlife Research Unit,
Rubenstein School of Environment and Natural Resources, University of Vermont,
Burlington, VT 05405, USA
tdonovan@uvm.edu

File list (files found within DataS1.zip)

- 'Code'
  - `Appendix-S3-Script.R`
  - `Simulation-Functions.R`
  - `Simulation-Script.R`

- 'Data'
  - 'appendix-results'
    - `amdata_object.RDS`
    - `bias_detection.RDS`
    - `bias_state.RDS`
    - `presence_warnings.RDS`
  - 'simulation-results'
    - `amdata_object.RDS`
    - `bias_detection.RDS`
    - `bias_state.RDS`
    - `presence_warnings.RDS`

Description

- 'Code' – folder containing three .R files, all three of which are heavily commented to be following along with by a user.
    - `Appendix-S3-Script.R` – an R script to replicate the simulation results contained in Appendix S3 (copies of which are provided in the Data folder 'appendix-results').
    - `Simulation-Functions.R` – an R file containing all functions required to run Appendix-S3-Script.R and Simulation-Script.R.
    - `Simulation-Script.R` – an R script to replicate the simulation results contained in the main body of the paper (copies of which are provided in the Data folder 'simulation-results').

- 'Data' – folder containing two folders of results
    - 'appendix-results' – a folder containing all data produced by the Appendix S3 simulation results (which can be reproduced using `Appendix-S3-Script.R`)
        - `amdata_object.RDS` – an RDS file containing an AMModels class 'amData' object which stores dynamics, encounter histories, data summaries, and parameter estimates from each of 100 replicates of all 192 appendix simulation scenarios.
        - `bias_detection.RDS` – an RDS file containing a data.table with dimensions 19,200 x 18 that stores parameter estimates from each of 100 replicates of all 192 appendix simulation scenarios, for the occupancy detection parameters *p11, p10*, and *b*. This object is used to produce plots of the bias of detection parameters with the function simBiasPlot() provided in `Simulation-Functions.R` and used in `Appendix-Script.R`.
        - `bias_state.RDS` – an RDS file containing a data.table with dimensions 19,200 x 18 that stores parameter estimates from each of 100 replicates of all 192 appendix simulation scenarios, for the occupancy state parameters psi (ψ), gamma (γ), and epsilon (ε). This object is used to produce plots of the bias of state parameters with the function simBiasPlot() provided in `Simulation-Functions.R` and used in `Appendix-Script.R`.
        - `presence_warnings.RDS` – an RDS file containing a data.table storing the scenario name ('scenario') and replicate number ('rep') of each scenario-replicate that received a warning during model fitting from the program PRESENCE. The column 'conv.warning' tracks whether this scenario-replicate received a convergence warning, and if so, at what value. The 'VC.warning' column tracks whether this scenario-replicate received a warning about the variance-covariance matrix. This object is used so that scenario-replicates that failed to converge may be removed from plots of parameter bias with the

function simBiasPlot() provided in `Simulation-Functions.R` and used in `Appendix-Script.R`.

- ‘simulation-results’ – a folder containing all data produced by the Appendix S3 simulation results (which can be reproduced using `Simulation-Script.R`)
  - `amdata_object.RDS` – an RDS file containing an AMModels class ‘amData’ object which stores dynamics, encounter histories, data summaries, and parameter estimates from each of 500 replicates of all 128 simulation scenarios.
  - `bias_detection.RDS` – an RDS file containing a data.table with dimensions 192,000 x 18 that stores parameter estimates from each of 500 replicates of all 128 simulation scenarios, for the occupancy detection parameters *p11, p10*, and *b*. This object is used to produce plots of the bias of detection parameters with the function simBiasPlot() provided in `Simulation-Functions.R` and used in `Simulation-Script.R`.
  - `bias_state.RDS` – an RDS file containing a data.table with dimensions 192,000 x 18 that stores parameter estimates from each of 500 replicates of all 128 simulation scenarios, for the occupancy state parameters psi (ψ), gamma (γ), and epsilon (ε). This object is used to produce plots of the bias of state parameters with the function simBiasPlot() provided in `Simulation-Functions.R` and used in `Simulation-Script.R`.
  - `presence_warnings.RDS` – an RDS file containing a data.table storing the scenario name (‘scenario’) and replicate number (‘rep’) of each scenario-replicate that received a warning during model fitting from the program PRESENCE. The column ‘conv.warning’ tracks whether this scenario-replicate received a convergence warning, and if so, at what value. The ‘VC.warning’ column tracks whether this scenario-replicate received a warning about the variance-covariance matrix. This object is used so that scenario-replicates that failed to converge may be removed from plots of parameter bias with the function simBiasPlot() provided in `Simulation-Functions.R` and used in `Simulation-Script.R`.
